# Quantitative Standardized Expansion Assay: An Artificial Intelligence-Powered Morphometric Description of Blastocyst Expansion and Zona Thinning Dynamics

**DOI:** 10.3390/life14111396

**Published:** 2024-10-30

**Authors:** Danilo Cimadomo, Samuele Trio, Tamara Canosi, Federica Innocenti, Gaia Saturno, Marilena Taggi, Daria Maria Soscia, Laura Albricci, Ben Kantor, Michael Dvorkin, Anna Svensson, Thomas Huang, Alberto Vaiarelli, Gianluca Gennarelli, Laura Rienzi

**Affiliations:** 1 IVIRMA Global Research Alliance, GENERA, Clinica Valle Giulia, 00197 Rome, Italy; federica.innocenti@ivirma.com (F.I.); gaia.saturno@generapma.it (G.S.); marilena.taggi@ivirma.com (M.T.); daria.soscia@generapma.it (D.M.S.); laura.albricci@generapma.it (L.A.); alberto.vaiarelli@gmail.com (A.V.); laura.rienzi@ivirma.com (L.R.); 2 IVIRMA Global Research Alliance, DEMETRA, 50141 Florence, Italy; samuele.trio@gmail.com; 3 Department of Biology and Biotechnology “Lazzaro Spallanzani”, University of Pavia, 27100 Pavia, Italy; tamara.canosi01@universitadipavia.it; 4 Department of Biomedicine and Prevention, University of Tor Vergata, 00128 Rome, Italy; 5 Fairtilty Ltd., Tel Aviv 6721508, Israel; ben@fairtility.com (B.K.); michael.dvorkin@fairtility.com (M.D.); anna.svensson@fairtility.com (A.S.); 6 Pacific In Vitro Fertilization Institute, Honolulu, HI 96826, USA; huangt@hawaii.edu; 7 John A. Burns School of Medicine, University of Hawaii at Manoa, Honolulu, HI 96826, USA; 8 Gynecology and Obstetrics 1U, Physiopathology of Reproduction and IVF Unit, Department of Surgical Sciences, S. Anna Hospital, University of Turin, 10126 Turin, Italy; gennarelligl@gmail.com; 9 IVIRMA Global Research Alliance, Livet, 10126 Turin, Italy; 10 Department of Biomolecular Sciences, University “Carlo Bo” of Urbino, 61029 Urbino, Italy

**Keywords:** blastocyst expansion, zona pellucida thinning, euploidy, live birth, artificial intelligence

## Abstract

Artificial intelligence applied to time-lapse microscopy may revolutionize embryo selection in IVF by automating data collection and standardizing the assessments. In this context, blastocyst expansion dynamics, although being associated with reproductive fitness, have been poorly studied. This retrospective study (N = 2184 blastocysts from 786 cycles) exploited both technologies to picture the association between embryo and inner-cell-mass (ICM) area in µm^2^, the ICM/Trophectoderm ratio, and the zona pellucida thickness in µm (zp-T) at sequential blastocyst expansion stages, with (i) euploidy and (ii) live-birth per transfer (N = 548 transfers). A quantitative-standardized-expansion-assay (qSEA) was also set-up; a novel approach involving automatic annotations of all expansion metrics every 30 min across 5 h following blastulation. Multivariate regressions and ROC curve analyses were conducted. Aneuploid blastocysts were slower, expanded less and showed thicker zp. The qSEA outlined faster and more consistent zp thinning processes among euploid blastocysts, being more or as effective as the embryologists in ranking euploid embryo as top-quality of their cohorts in 69% of the cases. The qSEA also outlined faster and more consistent blastocyst expansion and zp thinning dynamics among euploid implanted versus not implanted blastocysts, disagreeing with embryologists’ priority choice in about 50% of the cases. In conclusion, qSEA is a promising objective, quantitative, and user-friendly strategy to predict embryo competence that now deserves prospective validations.

## 1. Introduction

The main tasks in the IVF laboratory are to optimize the laboratory conditions and the performance of the embryologists, as well as to score, rank, and select embryos to maximize the possibilities to achieve a live birth (LB). An efficient embryo selection tool to identify reproductively competent embryos would significantly reduce the time-to-pregnancy by preventing unnecessary embryo transfers (ETs), with relevant economic and psychological implications [1]. Still, currently, static morphological embryo assessment is the most common selection scheme, especially in non-preimplantation genetic testing (PGT) cycles. However, morphological assessment is inconsistent across the embryologist community, especially across different IVF centers [2].

The introduction of new technologies, such as time-lapse microscopy (TLM) and artificial intelligence (AI) in clinical embryology, have provided novel and advanced information on human embryo development. TLM permits undisturbed culture conditions, allowing for continuous monitoring of embryo development dynamics. Several studies have leveraged TLM with the aim of identifying morphokinetic markers of embryonic competence [3]; however, to date, TLM-driven embryo selection has not been shown to be better than an embryologist’s assessment [4,5]. Nonetheless, the implementation of TLM to investigate human preimplantation development has provided embryologists with a powerful tool to precisely evaluate the timings of cellular divisions and detect abnormal dynamics that would be impossible to assess through static assessments (e.g., trichotomous cleavage or spontaneous blastocyst collapse) [6].

Lately, AI has been subject to several studies because it holds the potential to objectify and automate embryo assessment and enhance embryo selection by extracting relevant information from embryo microscopy images and videos. AI models aim at identifying embryos with the highest chance to implant in each cohort and provide embryologists with a score to prioritize them for transfer [7]. AI-based TLM assessment represents a powerful approach to studying embryo preimplantation development with countless possibilities.

In this context, blastocyst expansion represents an interesting feature to investigate. Indeed, it has been previously reported to be associated with embryo competence through both static and dynamic morphological assessments [8,9]. Notably, blastocyst expansion is a morphogenetic process common to several species [10], regulated by trophectoderm (TE) functional properties after embryonic genome activation. This phase is indeed very sensitive, being the first process occurring after inner cell mass (ICM) and TE differentiation. Several authors have indeed focused their attention on the clinical implications of blastocyst expansion, being mostly concordant in reporting a positive association [11,12,13,14].

Huang and colleagues designed a dynamic and standardized blastocyst expansion assessment scheme called quantitative standardized expansion assay (qSEA). This approach aimed at minimizing the variability across the operators in assessing blastocysts’ quality by focusing instead on their expansion at well-defined timepoints. Specifically, beginning at time of blastulation (tB) for 10 h at 2 h intervals, these authors measured the cross-sectional area of the embryo cavity and its surrounding TE in µm^2^ through the Embryoscope’s measurement tool. The data reported significantly associated with blastocyst competence, suggesting qSEA is a promising embryo selection tool that is less exposed to subjective evaluations and suitable for automation [9,15,16]. The recent implementation of AI-powered software for dynamic embryo assessment allowed us to slightly adapt the qSEA and enhance it with additional morphometric information (e.g., zona pellucida thickness). The aim of this retrospective study was to adopt an AI-powered version of the qSEA in a large dataset of 2184 biopsied blastocysts—of which 548 transferred—obtained during 786 PGT-A cycles. We assessed the association of the parameters extracted with this bioinformatic pipeline with embryo chromosomal competence (euploidy) and the reproductive competence (LB per euploid ET). In a clinical simulation, we also tested the putative value of this tool for embryo selection purposes.

## 2. Materials and Methods

### 2.1. Study Population and Study Design

This is a retrospective study including 2184 blastocysts obtained from 786 PGT-A cycles cultured in a time-lapse incubator (Embryoscope, Vitrolife, Gothenburg, Sweden) between January 2013 and December 2020 at a private IVF Center in Rome (IVIRMA Global Research Alliance, Genera, Clinica Valle Giulia). Patient characteristics are summarized in Supplemental Table S1. They were mostly advanced maternal age and poor prognosis women. Of the 2184 total blastocysts included in the study, 40.5% (886/2184) were euploid and the remaining 59.5% (1298/2184) were aneuploid. The aim of the study was to evaluate the putative association between an AI-powered morphometric and morphodynamic assessment of blastocyst expansion with their chromosomal (i.e., euploidy) and/or reproductive competence (i.e., LB after vitrified-warmed euploid single embryo transfer). The analysis was conducted at each specific blastulation timings (as described below), as well as using an adapted protocol of the qSEA described by Huang previously [9,15,16] (also detailed below).

Ethic committee approval was obtained for the retrospective analysis of pseudonymized videos and data aimed at identifying patient, cycle or embryo features associated with IVF efficacy and/or efficiency.

### 2.2. IVF and PGT-A Laboratory Protocols

Ovarian stimulation was conducted with a GnRH antagonist protocol and ovulation was triggered with either GnRH-agonist or hCG based on patient characteristics and the judgment of a gynaecologist. Oocyte retrieval was conducted 35 h after trigger, and ICSI was performed according to a previously detailed protocol [17]. A continuous single culture medium (CSCM, Irvine Scientific, Santa Ana, CA, USA) was adopted, with a medium refresh on Day 5 in case of extended culture to Days 6 or 7. No laser assisted zona pellucida (zp) drilling was applied on Day 3, and the embryos were left undisturbed within an intact zp throughout their development until the fully expanded blastocyst stage, when TE biopsy was conducted as thoroughly described previously [18]. The biopsied blastocysts were vitrified within 30 min from biopsy to prevent their re-expansion. Comprehensive chromosome testing (CCT) was conducted through qPCR or NGS at an external laboratory to report uniform aneuploidies [19,20]. Intermediate chromosomes copy numbers (ICN) were reported as either euploid or aneuploid [20]. Only vitrified-warmed single euploid blastocyst transfers were conducted with either a hormonal replacement therapy or a modified-natural endometrial preparation protocol. All transfers from the same oocyte retrieval were included. Blastocyst morphology was graded by the embryologists relying on Gardner’s scoring system [21] as excellent if AA; good if AB or BA; average if BB, AC, or CA; and low if CC, BC, or CB.

### 2.3. Blastocyst Expansion AI-Powered Morphometric and Morphodynamic Analysis

**Figure 1:**
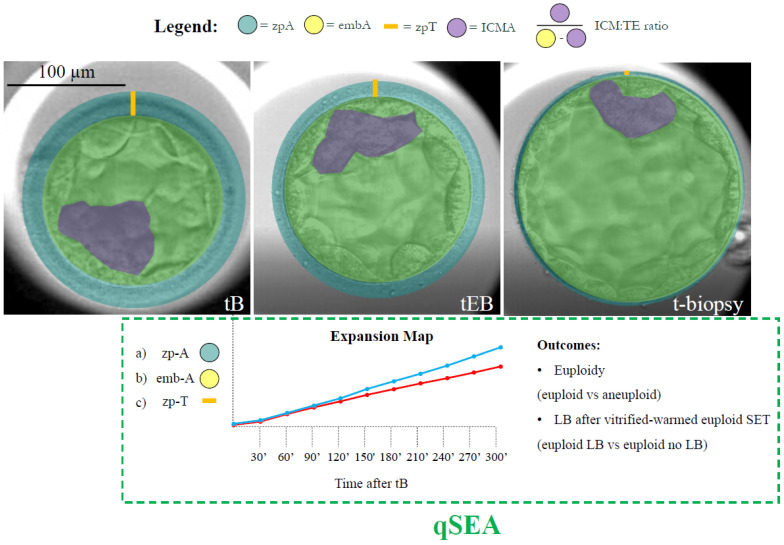
Definition of the features and timings under investigation. The AI-powered software CHLOE™ (Fairtility, Tel Aviv, Israel) was adopted to automatically annotate the time of blastulation (tB); the time of expanding blastocyst (tEB) (according to the definitions of the ESHRE TLT working group); and the time of biopsy (t-biopsy; i.e., the end of the video, when trophectoderm biopsy was performed) in hours post insemination (hpi). The same software annotated the area of embryo including the zp (zp-A; green circle); the area of the embryo proper (emb-A; yellow circle); the thickness of the zona pellucida (zp-T; calculated as the largest distance between the emb-A and the zp-A edges; orange line); the area of the ICM (ICM-A; purple shade); and the ratio between the area of ICM and the area of the trophectoderm (ICM/TE ratio). All of these metrics were calculated by the software at the median focal plane as the proportions of video frames occupied by each feature under investigation (single pixel = 300 µm; whole wells’ area = 90,000 µm^2^) at each blastulation timing. The quantitative standardized expansion assay (qSEA) was also automatically generated for each embryo by annotating the zp-A, emb-A, and the zp-T every 30 min across the 5 h following the tB. These data were then clustered according to blastocyst chromosomal constitution (euploid versus aneuploid) and reproductive competence (transferred euploid blastocysts that resulted in a LB versus transferred euploid blastocysts that did not result in a LB). This process generated six expansion maps (like the example with the blue and red lines for the two different outcomes) that were scrutinized to assess putative differences. Scale bar, 100 µm.

All Embryoscope videos were analysed through an AI-powered tool (CHLOE™, Fairtility, Tel Aviv, Israel) to automate, standardize, objectify, and quantify embryo assessment. CHLOE is an AI tool based on a convolutional neural network that automatically analyses embryo videos and annotates specific events of embryo development. Here we leveraged AI to annotate the time of starting blastulation (tSB); the time of blastulation (tB); the time of expanding blastocyst (tEB) (as described in [3,22]); and the time of biopsy (t-biopsy; i.e., the end of the video, when TE biopsy was performed) in hours post insemination (hpi). We also leveraged the AI to annotate the area of the embryo including the zp (zp-A); the area of the embryo proper (emb-A); the thickness of the zona pellucida (zp-T; calculated as the largest distance between the emb-A and the zp-A edges); the area of the ICM (ICM-A); and the ratio between the area of the ICM and the area of the TE (ICM/TE ratio) (Figure 1). All of these metrics were calculated by the software at the median focal plane as the proportions of video frames occupied by each feature under investigation (single pixel = 300 µm; whole wells’ area = 90,000 µm^2^) at each blastulation timing (Figure 1). Also, increases and decreases for all these measures were assessed between each blastulation timing and the following. All data were assessed for their association with euploidy and with LB among vitrified-warmed transferred euploid blastocysts.

### 2.4. AI-Powered qSEA

For zp-A, emb-A, and zp-T, the qSEA was also calculated by adapting Huang’s protocol [9,15,16]. In detail, these metrics were automatically annotated for each embryo by CHLOE every 30 min over the 5 h following the tB. The data were then clustered according to blastocyst chromosomal constitution (euploid versus aneuploid), and reproductive competence (transferred euploid blastocysts that resulted in a LB versus transferred euploid blastocysts that did not result in a LB). This generated six expansion maps that were scrutinized to assess putative differences in the expansion dynamics (Figure 1).

### 2.5. Clinical Simulations

Two simulations were conducted to assess the putative value of the qSEA had we used it clinically to prioritize blastocysts for transfer (as previously done for the iDAScore v1.0 [23]).

Among the 786 PGT-A cycles with ≥1 biopsied blastocyst, 352 obtained both euploid and aneuploid embryos. In these cycles, we could assess how often the embryologists and the qSEA were concordant and discordant in blastocyst ranking, and how often the highest ranked embryos were indeed euploid (the workflow is summarized in Supplemental Figure S1A).

Among the 237 PGT-A cycles with ≥2 euploid blastocysts, 216 underwent ≥1 ET (99 performed only 1 ET, 14 performed >1 ET always obtaining a LB, 50 performed >1 ET never obtaining a LB, and 53 performed >1 ET with both LB and implantation failures/miscarriages). In these cycles, we could assess how often the embryologists and the qSEA were concordant and discordant in ranking euploid blastocysts, and how often the highest ranked embryos indeed resulted in a LB (the workflow is summarized in Supplemental Figure S1B).

### 2.6. Statistical Analysis

The software SPSS version 29.0.1.0 (171) was used for statistics (IBM, Armonk, NY, USA). Continuous data are shown as mean ± standard deviation (SD). The normal (Gaussian) distribution of the data was assessed with a Shapiro–Wilk test and either *t*-test/ANOVA or Mann Whitney U/Kruskal Wallis tests were adopted to assess statistically significant differences. Categorical data are shown as ratios with percentages. Fisher’s exact or chi-squared tests were adopted to assess statistically significant differences. All putative associations of the data with euploidy among biopsied blastocysts, or reproductive competence (LB) among transferred euploid blastocysts, were confirmed with either linear or logistic regression analyses. The results were adjusted for relevant confounders identified among maternal age, BMI, previous conception(s) (no/yes), cause of infertility, duration of infertility, sperm quality, and blastocyst morphology.

## 3. Results

### 3.1. Aneuploid Blastocysts Were Slower, Expanded Less and Showed a Thicker Zona Pellucida with Respect to Euploid Embryos

The first analysis conducted was a comparison of all morphometric features at the tSB, tB, tEB, and t-biopsy among euploid and aneuploid blastocysts, as well as among transferred euploid blastocysts that resulted in a LB versus not (Table 1). The data were compared among the study groups and adjusted for confounders in case of significant associations at univariate analyses. The confounders were maternal age and blastocyst morphology for the euploidy outcome, and only blastocyst morphology for the LB outcome. Aneuploid blastocysts were slower than euploid over all blastulation timings from tSB to t-biopsy, while euploid reproductively incompetent blastocysts were slower than competent from tB onwards (Table 1). Aneuploid blastocysts, while starting from a larger expansion in terms of both zp-A and emb-A at the tB, showed both smaller zp-A and emb-A than euploid blastocysts at the t-biopsy (24,082 ± 5763 µm^2^ versus 25,438 ± 5968 µm^2^, adjusted-*p* < 0.01; and 23,612 ± 5960 µm^2^ versus 25,058 ± 6212 µm^2^, adjusted-*p* < 0.01) and expanded significantly less (zp-A t-biopsy/tEB: +38% ± 31% versus +47% ± 33%, adjusted-*p* < 0.01; zp-A t-biopsy/tB: +69% ± 39% versus +80% ± 41%, adjusted-*p* < 0.01; emb-A t-biopsy/tEB: +40% ± 34% versus +48% ± 36, adjusted-*p* < 0.01; emb-A t-biopsy/tB: +77% ± 44% versus +90% ± 47%, adjusted-*p* < 0.01) (Table 1; Supplemental Figure S2A,B). No association was reported between both parameters and the LB (Table 1). Also, the ICM-A and the ICM/TE ratio was comparable among euploids and aneuploids, as well as among euploids that resulted in a LB and euploids that failed to result in a LB (Table 1). Conversely, the zp-T was significantly thicker among aneuploid blastocysts versus euploids already at the tEB (12.9 ± 2.4 µm versus 12.6 ± 2.5 µm, adjusted-*p* = 0.01), a difference that became even more visible at the t-biopsy (8.1 ± 3.2 µm versus 7.1 µm ± 2.7, adjusted-*p* < 0.01), thereby outlining a significantly less consistent thinning (zp-T t-biopsy/tEB: −37% ± 24% versus −43% ± 22%, adjusted-*p* = 0.01; zp-T t-biopsy/tB: −50% ± 20% versus −55% ± 18%, adjusted-*p* < 0.01) (Table 1; Supplemental Figure S2C). Also in this case, no association was reported with the reproductive competence among transferred euploid blastocysts (Table 1).

When adjusting for maternal age, blastocyst morphological quality, and the zp-A, the zp-A ratio t-biopsy/tB showed a more significant association with euploid constitution than the zp-A per se, with a multivariate-OR 2.2, 95%CI 1.1–4.4, and an adjusted *p*-value = 0.02.

When adjusting for maternal age, blastocyst morphological quality and the emb-A, the emb-A ratio t-biopsy/tB showed a more significant association with euploid constitution than the emb-A per se, with a multivariate-OR 2.2, 95%CI 1.3–4.0, and an adjusted *p*-value < 0.01.

When adjusting for maternal age, blastocyst morphological quality and the zp-T ratio t-biopsy/tB, the zp-T showed a more significant association with euploid constitution than the ratio itself, with a multivariate-OR 0.92, 95%CI 0.86–0.98, and an adjusted *p*-value < 0.01.

### 3.2. The Zona Pellucida Thinning Process in the 5 h Following the tB Was More Substantial Among Euploid Blastocysts than Aneuploid

The definition of the t-biopsy is based on embryologists’ assessments, daily workload, and IVF laboratory logistics; therefore, it cannot be fully standardized. Conversely, the tB is specific to each embryo (i.e., “Time from insemination to formation of a full blastocyst; when the blastocoele filled the embryo with <10% increase in its diameter” according to [22] or “last frame before zona starts to thin” according to [3]), and it is automatically annotated by CHLOE. Moreover, blastocysts are never biopsied within 5 h following the tB, as they cannot achieve a suitable stage in such timeframe. These characteristics suggest that the qSEA is a more reliable and less biased strategy for assessing the association between blastocyst expansion dynamics and their competence.

When adopting the qSEA to compare aneuploid versus euploid blastocysts, no difference was reported for the emb-A and the zp-A (Supplemental Figures S3 and S4). Conversely, the zp-T showed a significantly slower and less consistent zona thinning among aneuploid blastocysts, which became significant already 150 min following the tB (even when adjusted for maternal age and blastocyte morphology) (Supplemental Figure S5A). The receiver operating characteristic (ROC) curve analysis showed an area under the curve (AUC) = 0.72, 95%CI 0.69–0.74, *p* < 0.01 for the prediction of euploidy based on the zp-T qSEA adjusted for maternal age. The same value was 0.75, 95%CI 0.73–0.77, *p* < 0.01 if considering the zp-T along with maternal age, blastocyst morphology, and the t-biopsy (Supplemental Figure S5B).

### 3.3. zp-A, emb-A, and zp-T qSEA Were Significantly Associated with Euploid Blastocysts’ Reproductive Competence

**Figure 2:**
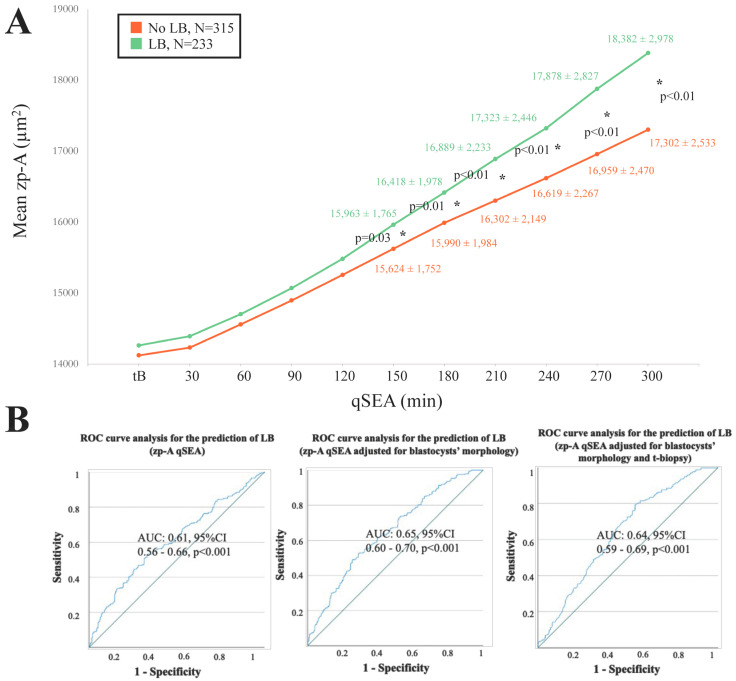
(**A**) The zp-A (area of the embryo including the zona pellucida in µm^2^) qSEA (quantitative standardized expansion assay) outlined a larger expansion among euploid blastocysts that resulted in a live birth (LB) (green line) versus euploid blastocysts that did not result in a LB (orange line), which became significant 150 min following the time of blastulation (tB). The stars (*) identify the significant datapoints showing the mean ± SD in the two groups at that timing. (**B**) Receiver Characteristics Operating (ROC) curve analyses outlined a significant association between the zp-A qSEA with a LB after euploid blastocyst transfer unadjusted, adjusted for blastocyst morphology, and adjusted for blastocyst morphology and time of biopsy (t-biopsy). AUC, area under the curve.

**Figure 3:**
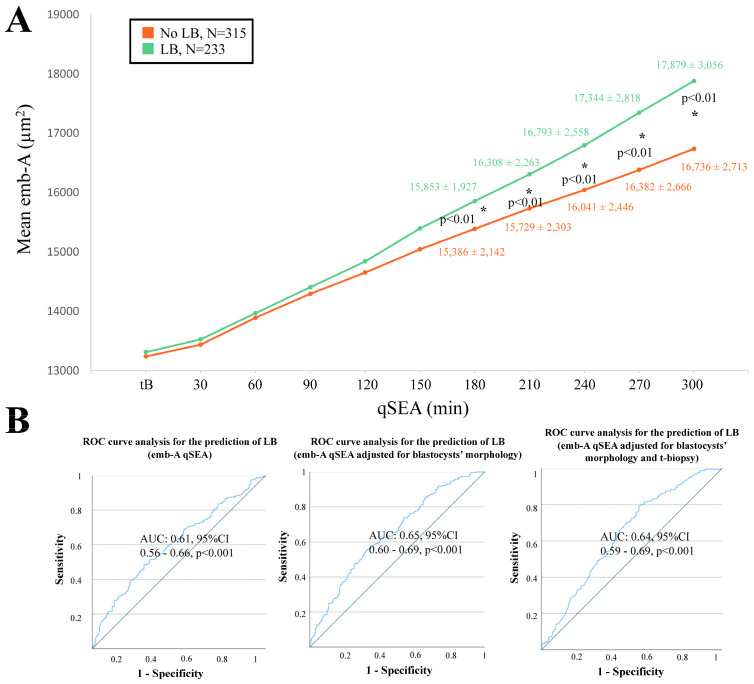
(**A**) The emb-A (area of the embryo proper in µm^2^) qSEA (quantitative standardized expansion assay) outlined a large expansion among euploid blastocysts that resulted in a live birth (LB) (green line) versus euploid blastocysts that did not result in a LB (orange line), which became significant already 180 min following the time of blastulation (tB). The stars (*) identify the significant datapoints showing the mean ± SD in the two groups at that timing. (**B**) Receiver Operating Characteristics (ROC) curve analyses outlined a significant association between the emb-A qSEA with a LB after euploid blastocyst transfer unadjusted, adjusted for blastocyst morphology, and adjusted for blastocyst morphology and time of biopsy (t-biopsy). AUC, area under the curve.

**Figure 4:**
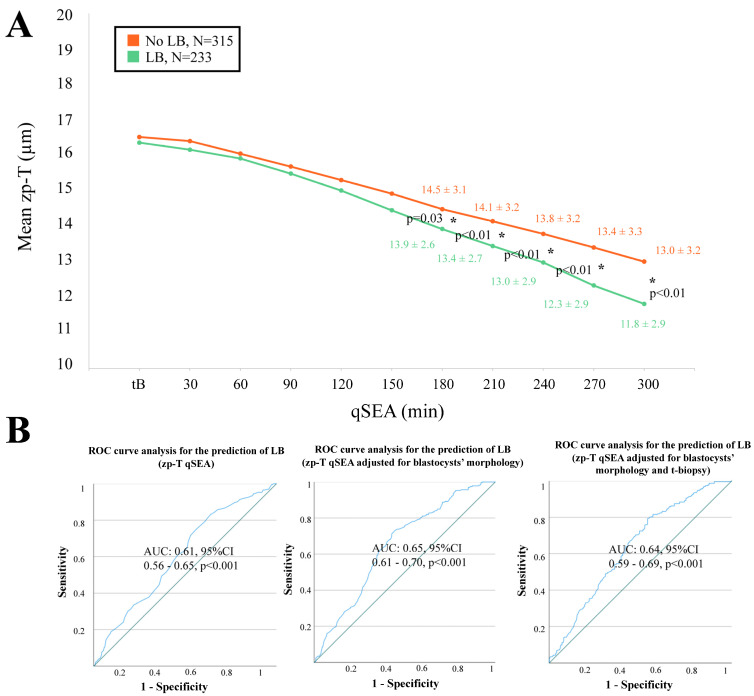
(**A**) The zp-T (thickness of the zona pellucida in µm) qSEA (quantitative standardized expansion assay) outlined a more consistent zona thinning among euploid blastocysts that resulted in a live birth (LB) (green line) versus euploid blastocysts that did not result in a LB (orange line), which became significant already 180 min following the time of blastulation (tB). The stars (*) identify the significant datapoints showing the mean ± SD in the two groups at that timing. (**B**) Receiver Characteristics Operating (ROC) curve analyses outlined a significant association between the zp-T qSEA with a LB after euploid blastocyst transfer unadjusted, adjusted for blastocyst morphology, and adjusted for blastocyst morphology and time of biopsy (t-biopsy). AUC, area under the curve.

When adopting the qSEA to compare euploid blastocysts resulting in a LB versus reproductively incompetent blastocysts, all three qSEA showed a significant association (Figure 2A, Figure 3A and Figure 4A). The zp-A qSEA outlined significant differences already after 150 min following the tB (Figure 2A), while the emb-A and the zp-T did so after 180 min (Figure 3A and Figure 4A). The ROC curve analyses showed AUCs to be almost superimposable for all three qSEA models, either per se (AUC = 0.61) or adjusted for confounders (AUC = 0.64–0.65) (Figure 2B and Figure 3B).

### 3.4. The zp-T qSEA Would Have Ranked a Euploid Blastocyst as Top Quality in Its Cohort in 57% of the Cycles with >1 Biopsied Blastocyst and Both Euploid and Aneuploid Embryos

As detailed previously, 352 cycles could be included in a clinical simulation to assess whether the zp-T qSEA (versus the embryologists) would have ranked a euploid blastocyst as top-quality within cohorts with >1 biopsied blastocyst and both euploid and aneuploid embryos (Supplemental Figure S1A). In 24% of the cases (N = 85/352), the embryologists and the zp-T qSEA would have agreed on the highest-ranked blastocyst; in 42% of the cases (N = 147/352), they would have disagreed; and in the remaining 34% of the cases (N = 120/352), the embryologists ranked more than one blastocyst as top-quality in their cohort (Supplemental Figure S6). Overall, the embryologists’ highest ranked blastocysts were: (i) euploid in 45% of the cases (N = 159/352) versus 57% for the zp-T qSEA (N = 199/352); (ii) aneuploid in 25% of the cases (N = 89/352) versus 43% for the zp-T qSEA (N = 153/352); and (iii) both euploid and aneuploid in 30% of the cases (N = 104/352) versus never for the zp-T qSEA (Supplemental Figure S6). In fact, it is highly unlikely that two blastocysts would be equally ranked by the qSEA, being that this strategy is based on highly specific and objective measurements.

### 3.5. In 69% of the Cycles with >1 Biopsied Blastocyst and Both Euploid and Aneuploid Embryos, the zp-T qSEA Ranking Would Be Equal or Better than Embryologist Rankings

When embryologists and the zp-T qSEA were compared for their putative effectiveness in blindly ranking euploid blastocysts as top quality within their cohorts: (i) they would have been equally effective in 43% of the cases (being either both wrong [N = 47/352, 13%] or both right [N = 104/352, 30%]); (ii) the embryologists would have been more effective in 15% of the cases (N = 52/352); (iii) the zp-T qSEA would have been more effective in 7% of the cases (N = 26/352); (iv) the zp-T qSEA would have ranked a euploid on top while the embryologists both a euploid and an aneuploid in 19% of the cases (N = 66/352); and (v) the zp-T qSEA would have ranked an aneuploid on top while the embryologists both a euploid and an aneuploid in 16% of the cases (N = 57/352) (Supplemental Figure S6). The raw data of this clinical simulation are provided in Supplemental Table S2.

### 3.6. In 46% of the Cases, the zp-A and emb-A qSEAs Would Have Disagreed with the Embryologists in Prioritizing Euploid Blastocysts for Transfer; The zp-T qSEA, Instead, Would Have Disagreed in 60% of the Cases

As detailed previously, 216 cycles could be included in a clinical simulation to assess the zp-A, emb-A, and zp-T qSEAs performance versus the embryologists in prioritizing euploid blastocysts for transfer within cohorts with ≥2 euploid blastocysts (Figure 2B). Specifically, in 99 cycles there were ≥2 euploid blastocysts but only 1 had been transferred, in 14 cycles there were ≥2 euploid blastocysts and all resulted in a LB, in 50 cycles there were ≥2 euploid blastocysts and all did not result in a LB, and in 53 cycles there were ≥2 euploid blastocysts resulting in both LBs and implantation failures/miscarriages. In 54% of the cases (N = 116/216), the embryologists and the zp-A or the emb-A qSEA would have agreed on euploid blastocyst prioritization for transfer, while in 46% of the cases (N = 100/216) they would have disagreed (Supplemental Figure S7A,B). The same results for the zp-T qSEA would have been 40% (N = 86/216) and 60% (N = 130/216), respectively (Supplemental Figure S7C). In terms of clinical effectiveness, the embryologists prioritized for transfer a competent euploid blastocyst in 47% of the cases (N = 102/216). In this scenario, the zp-A and the emb-A qSEA choice would have been correct in 38% of the cases (N = 82/216 and 81/216, respectively). However, this outcome may improve up to 61%, because in 23% of the cases (N = 50/216 for both the zp-A and emb-A) the euploid blastocysts with the highest priority according to the zp-A and emb-A qSEA had not been transferred yet (Supplemental Figure S7A,B). Regarding the zp-T qSEA, instead, its choice would have been correct in 27% of the cases (N = 59/216). However, this outcome may improve up to 63%, because in 36% of the cases (N = 78/216) the euploid blastocysts with the highest priority according to the zp-T qSEA had not been transferred yet (Supplemental Figure S7C). The raw data of this clinical simulation are provided in Supplemental Figure S7A–C and in Supplemental Tables S3–S5).

## 4. Discussion

Blastocyst morphological quality is significantly associated with both the chromosomal and reproductive competence of embryos [24]; however, it cannot be used alone to accurately predict these outcomes. Currently, blastocyst morphological quality is primarily assessed in IVF laboratories using grading systems such as the Gardner system [21]. Nonetheless, these grading systems are subjective and often vary among clinical embryologists, particularly when they work at different IVF centers or have undergone diverse training programs [2]. To address these limitations, AI-powered tools have been developed to analyze static images or time-lapse videos of embryo development. These tools aim to objectively assess embryo quality and rank embryos within a cohort based on a standardized scoring system. Typically, a higher score indicates a greater likelihood that the embryo is euploid and/or will successfully implant after embryo transfer [7,25]. In this study, we evaluated a qSEA model, determined its association with embryo euploidy and its potential to predict live birth outcomes. Both a comprehensive analysis and an intra-cohort clinical simulation were performed to test its effectiveness.

### 4.1. Clinical Implications of the Evidence Produced in This Study

Blastocyst expansion dynamics might provide relevant information on embryo reproductive competence. Indeed, several studies suggested that, among blastocyst morphological characteristics, expansion represents an intriguing feature to consider when ranking embryos in terms of priority for transfer [8,26,27] in both conventional and PGT-A cycles. Based on these preliminary data from the literature, we aimed at producing objective, morphometric and reproducible information on the expansion process, to reliably assess a putative association between these embryo-specific dynamics and its chromosomal (i.e., euploidy) and/or reproductive competence (i.e., LB after euploid blastocyst transfer). Huang’s former positive experience with the qSEA [9,15,16], and the possibility of integrating this method with AI-standardized and -powered analyses and PGT-A, incited us to test it for its association with blastocyst competence in our setting and patient population. Our study improved Huang’s previous experience through a large sample size (N = 2184), the use of PGT-A to assess the chromosomal constitution of all blastocysts, and the adoption of a TE biopsy protocol that does not entail assisted hatching on Day 3, thereby not affecting the process of blastocyst physiological expansion.

Besides confirming that aneuploid and euploid reproductively incompetent embryos are generally slower in reaching all blastulation timings milestones from tSB to t-biopsy [28,29], our data have highlighted an association between euploidy and larger zp-A and emb-A, as well as thinner zp at t-biopsy. Also, the zp-A and emb-A ratio t-biopsy/tB and the zp-T ratio t-biopsy/tB outlined larger expansion and more consistent zona thinning among euploid blastocysts versus aneuploid. Possibly, different issues—either chromosomal, metabolic, or structural—affect embryo timeliness in setting up TE epithelium architecture. This in turn could affect its possibility to cope with the increased hydrostatic pressure, essential for the expansion process. This hypothesis is consistent with a recent study [30], where the authors attempted to define to what extent blastocyst expansion dynamics could be affected by different classes of aneuploidies. Interestingly, all classes of aneuploidies, except for trisomies, significantly impaired the expansion process compared to euploid blastocysts. As has already been shown across several studies in the literature, TE characteristics seem more relevant than ICM in terms of association with embryo reproductive competence [8,31,32]. Indeed, the ICM-A and the ICM/TE ratio showed no association with all outcomes under investigation. This evidence might mirror the predominant role of the TE over the ICM in the process of implantation when it must establish a timely and effective dialogue with the endometrium.

Although blastocyst static morphometric evaluation before vitrification or biopsy in the absence of time-lapse incubators might also be clinically relevant, it is inherently biased by logistic issues, like daily workload, lab schedule, and embryologist decisions. The qSEA instead represents a less biased and more embryo-centred assessment, because the tB is reached by all useable blastocysts, regardless of their developmental pace, and more than 5 h always elapse from this timing and biopsy. Interestingly, consistent with Huang’s previous experience, the expansion maps showed significant associations between the zp-A, emb-A, and zp-T qSEAs and euploid blastocyst reproductive competence, even when adjusted for blastocyst morphology and t-biopsy (i.e., the main embryological confounders upon euploid embryo implantation potential), already 150–180 min following the tB. The zp-T qSEA outlined significant associations with the euploidy as well. Although this overall association already has important implications in terms of basic knowledge (which we will discuss below), we were interested in defining the putative clinical relevance of qSEA had we used it clinically in our setting. In fact, to improve the performance of embryologists with embryo ranking and prioritization for transfer, the information provided by this tool (as any other embryo selection tool) must be clinically effective when used within a given cohort of embryos produced by a couple. To this end, we conducted two clinical simulations (as previously done for iDAScore v1.0 [23]): (i) among cycles with ≥2 biopsied blastocysts, of which both euploid and aneuploid, to test zp-T qSEA euploidy prediction; and (ii) among cycles ≥2 euploid blastocysts, of which ≥1 transferred, to test all qSEA LB prediction. Notably, the zp-T qSEA would have disagreed with the embryologists in ranking embryos in 42% of cases, while it would have represented a swing vote between two blastocysts equally ranked on top by the operators in a further 34% of the cases. This information is interesting, especially considering that the zp-T qSEA ranked euploid blastocysts on top in 57% of the cases and it would have been right with the embryologists being either wrong or uncertain in 26% of the cases. In our population of advanced maternal age women, these data are even more intriguing. When tested for its prediction of a LB in the context of euploid blastocyst transfers among cohorts with ≥2 euploid embryos, the zp-A and emb-A qSEAs disagreed with the embryologists in embryo prioritization in 46% of the cases, a rate that increased to 60% for the zp-T qSEA. Again, this is interesting because it outlines a scenario where there is room for improving embryo selection when adopting this tool with respect to embryologist evaluations. In 38% and 27% of the cases, the zp-A or emb-A qSEAs and zp-T qSEA, respectively, would have correctly prioritized for transfer a competent euploid blastocyst. Both these rates may significantly increase in the future considering that in 23% and 36% of the cases, the blastocysts that would have been chosen based on the zp-A or emb-A qSEAs and the zp-T qSEA, respectively, had not been transferred yet. In the case of a positive outcome after transfer, these instances will increase the prevalence of events where the qSEA would have outperformed the embryologists (currently 8% and 10% for the zp-A or emb-A qSEAs and zp-T qSEA, respectively). Only a future RCT comparing embryologist rankings versus qSEA rankings to prioritize the first blastocyst for transfer in the context of either conventional or PGT-A cycles would ultimately unveil the true clinical benefit of this tool. Until then, these preliminary data should be considered only promising.

The main limitation of this study is inherent to its retrospective nature. Moreover, this study represents the second experience worldwide with the qSEA; confirmatory evidence should therefore be derived from further investigation at other centres and in other settings to validate these findings.

### 4.2. Basic Science Data Supporting the Evidence Produced in This Study

The cavitation and expansion processes are regulated by several genes, including E-cadherin, catenin, tight junction proteins, and the sodium–potassium ATPase transport system [33]. The initiation of blastulation is characterized by the formation of a small cavity or multiple microlumens eventually merging. A study on mice has proposed that vesicles or vacuoles are involved in blastocoel formation as they are released by exocytosis at the level of the basal membrane, thereby producing the microlumens [34]. More recently, another group proposed a “hydro-osmotic coarsening theory” suggesting that osmotic pressure at the level of the intercellular cell junctions transiently disrupts cell–cell contact, forming numerous small lumens [35]. These lumens then undergo intercellular ion and fluid exchange, eventually coalescing into a single lumen, i.e., the blastocoel cavity. After the initial microlumen formation, further expansion results from the active Na/K-ATPase transport mechanism located on the basolateral surface of the TE epithelium. This generates a sodium gradient, in turn ensuring water influx from the external environment into the microlumen [36,37]. Along with the osmotic gradient, water channels known as aquaporins (AQPs) facilitate water influx through the outer cells. Various AQP isoforms are expressed during preimplantation development in mice, and each isoform may play a unique role because of their temporally and spatially distinct expression patterns [38]. Simultaneously, the adherens junction, tight junction, desmosomes, and gap junction, form a seal between the TE cells, which are pivotal for fluid intake and blastocoel formation and expansion [33]. From a basic cellular perspective, the chromosome instability at the origin of aneuploidies seems to universally compromise cellular fitness [39,40,41]. It is reasonable to hypothesize that the resulting imbalances in both transcription and translation can affect both the cellularity and function of the emerging TE to produce and maintain the blastocoel fluid that will be critical both for hatching and for productive, sustained invasion of the endometrium. Transcription defects affecting the various players involved in these processes, as well as external environmental insults may impact the cavitation process and alter the viscoelastic properties of the blastocyst [42,43,44]. Throughout blastocyst expansion, the blastocyst applies hydrostatic mechanical pressure on the zp, progressively leading to its thinning up to the creation of a nick [45]. Following, expansion allows the embryo to hatch and possibly implant. In mice, this hatching process is facilitated by the timely secretion of implantation-related molecules, such as cytokines [45] and zonalytic proteases, like strypsin, hepsin, and cathepsin [46] by both TE and endometrial cells [45,47]. Proteases might be involved in this process in humans as well, according to recent data [48]. In general, the extent of zp thinning might mirror the timeliness of these finely regulated spatiotemporal processes, providing relevant information to predict blastocysts reproductive potential in mammals, including humans [49]. Previous studies have suggested that zp-T and zp-T variation may be useful additional parameters for embryo assessment, in addition to conventional morphological grading. Indeed, embryos with a thinner zp or greater zp-T variation showed a greater chance of implantation and evolution in an ongoing pregnancy than embryos with thicker zp [50,51,52,53]. In general, a thinner zp, even if localized just to some areas, might facilitate hatching, as well as the transportation and release of lytic enzymes and/or messengers (e.g., miRNAs), ultimately supporting implantation [50,54]. In summary, the process of blastulation, expansion, and hatching certainly deserve future deeper biomechanical investigations, as their characterization holds potential for generating relevant evidence for embryo selection purposes as well.

### 4.3. Future Perspectives of Molecular Investigations

In the future, integrating molecular data, particularly transcriptomic analyses, with genomic and chromosomal assessments from a single biopsy may significantly enhance our understanding of early embryonic development and implantation processes [55]. Current clinical investigations still fail to capture the complete picture of embryonic competence. In-depth transcriptomic data could identify molecular markers that explain why certain embryos exhibit varying developmental rates, distinct levels of blastocyst expansion, and heterogeneous overall morphological quality, despite being derived from the same cohort of oocytes and exposed to the same in vitro culture conditions. These differences in gene expression may reveal underlying metabolic, cellular, or epigenetic factors with significant implications for embryo selection [56,57].

## 5. Conclusions

Blastocyst expansion dynamics, timing, and morphometrics measured by AI provide objective and quantitative data related to embryo competence. qSEA is a promising, user-friendly, and easily applicable strategy that deserves further evaluation. Basic research into the mechanisms controlling blastocyst expansion is also warranted.

## Figures and Tables

**Table 1:** Summary of the main results. Area of the zona pellucida (zp-A), area of the embryo proper (emb-A), thickness of the zona pellucida (zp-T), are of the inner cell mass (ICM-A), ratio between ICM and trophectoderm (ICM/TE ratio) at the time of blastulation (tB), at the time of blastocyst expansion (tEB) and at the time of biopsy (t-biopsy), between aneuploid versus euploid blastocysts, and between euploid not resulted and resulted in a live birth (LB). Also, the increase in all areas and the decrease in zp-T throughout consecutive timings and from tB to t-biopsy were reported and compared across the study groups.

	Aneuploid N = 1298	Euploid N = 886	*p*-Value	Euploid–No LB N = 315	Euploid–LB N = 233	*p*-Value
**tSB**, mean ± SD, hpi **tB**, mean ± SD, hpi **tEB**, mean ± SD, hpi **t-biopsy**, mean ± SD, hpi	102.5 ± 10.5 113.0 ± 12.7 120.9 ± 14.5 136.0 ± 15.2	100.5 ± 9.6 109.7 ± 11.1 115.9 ± 12.0 131.6 ± 13.7	*p* < 0.01 *p* < 0.01 *p* < 0.01 *p* < 0.01	100.3 ± 9.7 109.9 ± 11.2 116.7 ± 12.4 132.4 ± 13.9	99.0 ± 9.8 107.5 ± 10.9 112.6 ± 11.2 127.1 ± 12.5	*p* = 0.10 *p* = 0.01 *p* < 0.01 *p* < 0.01
**zp-A at tB**, mean ± SD, µm^2^	**14,288 ± 1257**	**14,168 ± 1119**	*p* = 0.02 *	14,124 ± 1071	14,263 ± 1231	*p* = 0.16
**zp-A at tEB**, mean ± SD, µm^2^	17,435 ± 1828	17,417 ± 1955	*p* = 0.82	17,482 ± 2234	17,542 ± 1835	*p* = 0.74
**Ratio tEB/tB**, mean ± SD	+22% ± 10%	+23% ± 12%	*p* = 0.74 *	+24% ± 14%	+23% ± 9%	*p* = 0.48
**zp-A at t-biopsy**, mean ± SD, µm^2^	**24,082 ± 5763**	**25,438 ± 5968**	*p* < 0.01 *	25,141 ± 5873	25,790 ± 6159	*p* = 0.21
**Ratio t-biopsy/tEB**, mean ± SD	**+38% ± 31%**	**+47% ± 33%**	*p* < 0.01 *	+45% ± 33%	+47% ± 33%	*p* = 0.35
**Ratio t-biopsy/tB**, mean ± SD	**+69% ± 39%**	**+80% ± 41%**	*p* < 0.01 *	+79% ± 42%	+81% ± 42%	*p* = 0.47
**emb-A at tB**, mean ± SD, µm^2^	**13,349 ± 1196**	**13,249 ± 1121**	*p* = 0.03 *	13,235 ± 1107	13,309 ± 1121	*p* = 0.44
**emb-A at tEB**, mean ± SD, µm^2^	16,900 ± 1867	16,922 ± 1986	*p* = 0.79	16,996 ± 2274	17,030 ± 1757	*p* = 0.85
**Ratio tEB/tB**, mean ± SD	+27% ± 11%	+28% ± 13%	*p* = 0.28 *	+29% ± 16%	+28% ± 10%	*p* = 0.64
**emb-A at t-biopsy**, mean ± SD, µm^2^	**23,612 ± 5960**	**25,058 ± 6212**	*p* < 0.01 *	24,694 ± 6169	25,512 ± 6299	*p* = 0.13
**Ratio t-biopsy/tEB**, mean ± SD	**+40% ± 34%**	**+48% ± 36%**	*p* < 0.01 *	+46% ± 35%	+50% ± 34%	*p* = 0.22
**Ratio t-biopsy/tB**, mean ± SD	**+77% ± 44%**	**+90% ± 47%**	*p* < 0.01 *	+87% ± 48%	+92% ± 46%	*p* = 0.26
**zp-T at tB**, mean ± SD, µm	16.4 ± 2.9	16.2 ± 2.9	*p* = 0.25	16.5 ± 3.0	16.3 ± 3.1	*p* = 0.55
**zp-T at tEB**, mean ± SD, µm	**12.9 ± 2.4**	**12.6 ± 2.5**	*p* = 0.01 *	12.9 ± 2.5	12.8 ± 2.5	*p* = 0.54
**Ratio tEB/tB**, mean ± SD	−21% ± 11%	−22% ± 11%	*p* = 0.53 *	−21% ± 11%	−22% ± 10%	*p* = 0.90
**zp-T at t-biopsy**, mean ± SD, µm	**8.1 ± 3.2**	**7.1 ± 2.7**	*p* < 0.01 *	7.3 ± 2.9	6.9 ± 2.5	*p* = 0.11
**Ratio t-biopsy/tEB**, mean ± SD	**−37% ± 24%**	**−43% ± 22%**	*p* = 0.01 *	−43% ± 22%	−44% ± 20%	*p* = 0.24
**Ratio t-biopsy/tB**, mean ± SD	**−50% ± 20%**	**−55% ± 18%**	*p* < 0.01 *	−55% ± 18%	−57% ± 16%	*p* = 0.21
**ICM-A at tB**, mean ± SD, µm^2^	3458 ± 905	3414 ± 902	*p* = 0.27	3425 ± 953	3434 ± 852	*p* = 0.91
**ICM-A at tEB**, mean ± SD, µm^2^	3497 ± 1047	3460 ± 1066	*p* = 0.43	3414 ± 1078	3468 ± 984	*p* = 0.55
**Ratio tEB/tB**, mean ± SD	+5% ± 35%	+5% ± 33%	*p* = 0.99	+4% ± 33%	+3% ± 25%	*p* = 0.65
**ICM-A at t-biopsy**, mean ± SD, µm^2^	3804 ± 1471	3727 ± 1469	*p* = 0.31	3800 ± 1547	3541 ± 1212	*p* = 0.08
**Ratio t-biopsy/tEB**, mean ± SD	+11% ± 50%	+10% ± 45%	*p* = 0.42	+13% ± 46%	+4% ± 36%	*p* = 0.17 **
**Ratio t-biopsy/tB**, mean ± SD	+12% ± 49%	+12% ± 51%	*p* = 0.97	+16% ± 56%	+6% ± 45%	*p* = 0.08
**ICM/TE ratio at tB**, mean ± SD	26% ± 7%	26% ± 7%	*p* = 0.61	26% ± 7%	26% ± 7%	*p* = 0.95
**ICM/TE ratio at tEB**, mean ± SD	21% ± 6%	21% ± 6%	*p* = 0.36	20% ± 6%	20% ± 6%	*p* = 0.50
**ICM/TE ratio at t-biopsy**, mean ± SD	17% ± 8%	16% ± 7%	*p* = 0.97 *	16% ± 7%	15% ± 6%	*p* = 0.47 **

* adjusted for maternal age and blastocyst morphological quality. ** adjusted for blastocyst morphological quality.

## Data Availability

The data underlying this article are available in the article and in its online Supplementary Materials.

## Supplementary Materials

The following supporting information can be downloaded at: https://www.mdpi.com/article/10.3390/life14111396/s1, Supplemental Figure S1. Study Flowchart. The figure summarizes the study flowchart and the number of preimplantation genetic testing for aneuploidies (PGT-A) cycles that could be included in the clinical simulations for euploidy (A) and live birth (LB) (B) prediction. Supplemental Figure S2. Euploid blastocysts (green boxplots) expand more (larger zp-A and emb-A, respectively area of the embryo in µm^2^ with or without the zona pellucida) between the time of blastulation (tB) and the time of biopsy (t-biopsy) and undergo a more consistent zona pellucida thinning (zp-T, thickness of the ZP in µm) than aneuploid blastocysts (red boxplots). Supplemental Figure S3. (A) The zp-A (area of the embryo including the zona pellucida in µm^2^) qSEA (quantitative standardized expansion assay) did not outline any significant difference between euploid (green line) and aneuploid (red line) blastocysts. Supplemental Figure S4. (A) The emb-A (area of the embryo proper in µm^2^) qSEA (quantitative standardized expansion assay) did not outline any significant difference between euploid (green line) and aneuploid (red line) blastocysts. Supplemental Figure S5. (A) The zp-T (thickness of the zona pellucida in µm) qSEA (quantitative standardized expansion assay) outlined a more consistent zona thinning among euploid blastocysts (green line) versus aneuploid blastocysts (red line), that became significant already 150 min following the time of blastulation (tB). The stars (*) identify the significant datapoints showing the mean ± SD in the two groups at that timing. (B) Receiver Characteristics Operating (ROC) curve analyses outlined a significant association between the zp-T qSEA with euploidy when adjusted only for maternal age, for maternal age blastocysts’ morphology, as well as for maternal age, blastocysts’ morphology and time of biopsy (t-biopsy). AUC, area under the curve. Supplemental Figure S6. Outcomes of a clinical simulation to assess zp-T (zona pellucida thickness in µm) qSEA (quantitative standardized expansion assay) performance versus the embryologists (E) in ranking euploid blastocysts on top among cohorts with both euploid and aneuploid siblings. Ranking concordance was not assessable any time ≥2 embryos were ranked on top by the embryologists, among which one was the zp-T qSEA highest ranked blastocyst (black bar of the histogram). Effectiveness in prioritizing the euploid blastocyst was not assessable any time ≥2 embryos were ranked on top by the embryologists, of which ≥1 was euploid and ≥1 was aneuploid (dark grey section of the pie chart; blue and brown bars of the histogram). Raw data are shown in Supplemental Table S2. Supplemental Figure S7. Outcomes of a clinical simulation to assess (A) zp-A (area of the embryo including the zona pellucida in µm^2^) qSEA (quantitative standardized expansion assay), (B) emb-A (area of the embryo proper in µm^2^) qSEA, and (C) zp-T (zona pellucida thickness in µm) qSEA performance versus the embryologists (E) in ranking euploid blastocysts resulting a live birth (LB) on top among cohorts with ≥2 euploid siblings. Effectiveness in prioritizing the competent euploid blastocyst was not assessable any time the highest ranked embryo according to the qSEA has not been transferred yet (dark grey sections of the pie charts; dark grey bars of the histograms). Raw data are shown in Supplemental Tables S3–S5. Supplemental Table S1. Patients’ characteristics. Supplemental Table S2. Distribution of the PGT-A cycles in the clinical simulation for the comparison of embryologists’ (E) versus zp-T qSEA’s performance in ranking euploid blastocysts on top. The results are summarized in Supplementary Figure S3. * ≥2 embryos ranked on top according to the embryologists, that included also the embryo ranked on top according to the zp-T qSEA. ^ ≥2 embryos ranked on top according to the embryologists, of which ≥1 euploid and ≥1 aneuploid. Supplemental Table S3. Distribution of the PGT-A cycles in the clinical simulation for the comparison of embryologists’ (E) versus zp-A qSEA’s performance in ranking euploid blastocysts that resulted in a live birth (LB) on top. The results are summarized in Supplementary Figure S4A. Supplemental Table S4. Distribution of the PGT-A cycles in the clinical simulation for the comparison of embryologists’ (E) versus emb-A qSEA’s performance in ranking euploid blastocysts that resulted in a live birth (LB) on top. The results are summarized in Supplementary Figure S4B. Supplemental Table S5. Distribution of the PGT-A cycles in the clinical simulation for the comparison of embryologists’ (E) versus zp-T qSEA’s performance in ranking euploid blastocysts that resulted in a live birth (LB) on top. The results are summarized in Supplementary Figure S4C.

## Abbreviations

IVF, in vitro fertilization; LB, live birth; ET, embryo transfer; PGT-A, preimplantation genetic testing for aneuplodies; TLM, time lapse microscopy; AI, artificial intelligence; ICM, inner cell mass; qSEA, quantitative standardized expansion assay; GnRH, gonadotrophins releasing hormone; hCG, human chorionic gonadotrophin; zp, zona pellucida; CCT, comprehensive chromosome testing; qPCR, quantitative polymerase chain reaction; NGS, next generation sequencing; ICN, intermediate copy number; tSB, time of starting blastulation; tB, time of blastulation; tEB, time of expanding blastocyst; t-biopsy, time of biopsy; TE, trophectoderm; zp-A, are of the zona pellucida; zq-T, thickness of the zona pellucida; emb-A, area of the embryo; ICM-A, area of the inner cell mass; ICM:TE, ratio between the area of the inner cell mass and the area of the trophectoderm; BMI, body mass index; ROC, receiver operating characteristic; AUC, area under the curve.
