# Supplementary material for: Quantitative Standardized Expansion Assay: An Artificial Intelligence-Powered Morphometric Description of Blastocyst Expansion and Zona Thinning Dynamics
Source: Life (Basel). 2024 Oct 30;14(11):1396. doi: 10.3390/life14111396 (PMC11595650; doi:10.3390/life14111396)
Supplement: Supplementary file 1 [file life-14-01396-s001.zip › Figure S1.pdf]

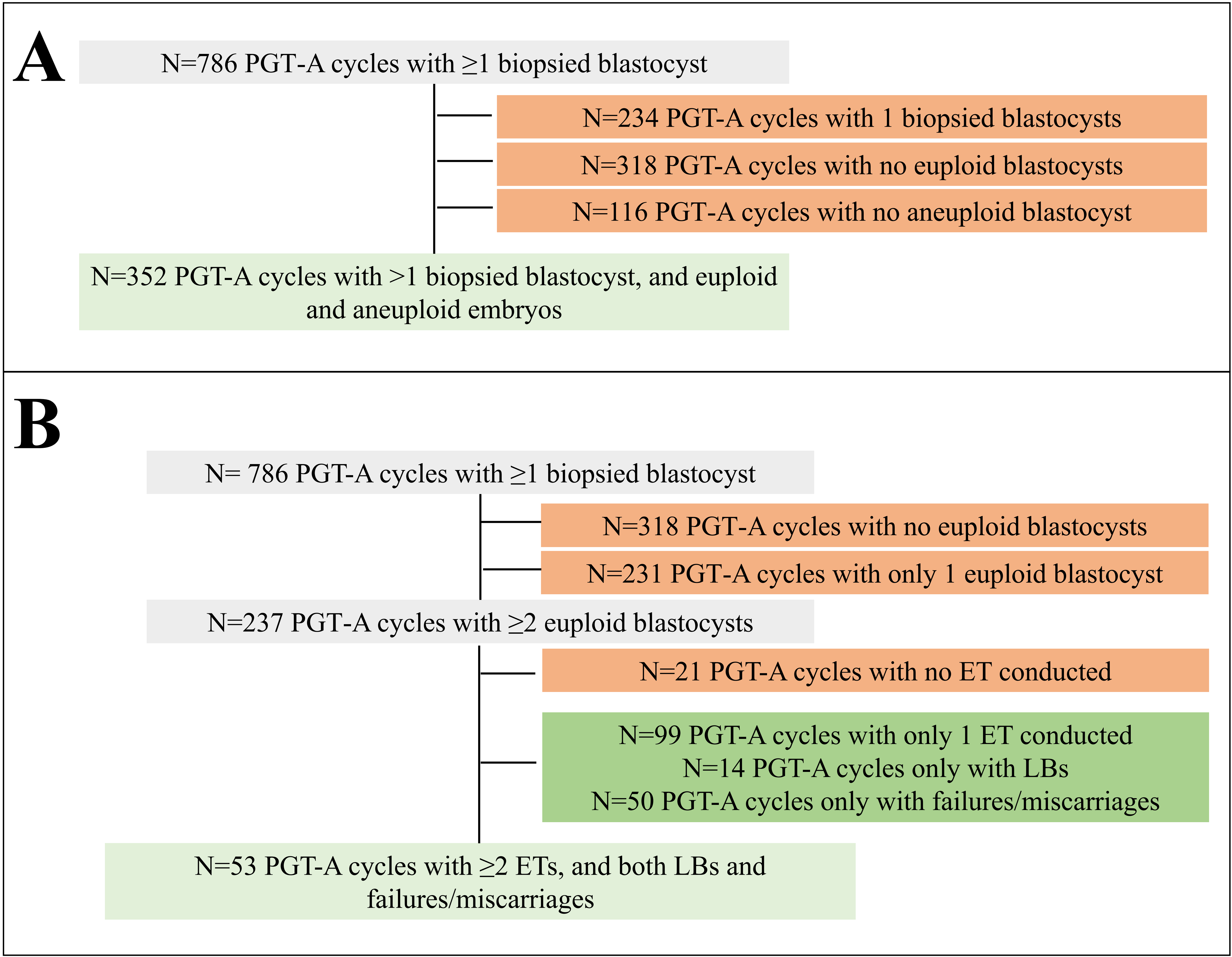
A
N=786 PGT-A cycles with ≥1 biopsied blastocyst
N=234 PGT-A cycles with 1 biopsied blastocysts
N=318 PGT-A cycles with no euploid blastocysts
N=116 PGT-A cycles with no aneuploid blastocyst
N=352 PGT-A cycles with >1 biopsied blastocyst, and euploid and aneuploid embryos
B
N= 786 PGT-A cycles with ≥1 biopsied blastocyst
N=318 PGT-A cycles with no euploid blastocysts
N=231 PGT-A cycles with only 1 euploid blastocyst
N=237 PGT-A cycles with ≥2 euploid blastocysts
N=21 PGT-A cycles with no ET conducted
N=99 PGT-A cycles with only 1 ET conducted
N=14 PGT-A cycles only with LBs
N=50 PGT-A cycles only with failures/miscarriages
N=53 PGT-A cycles with ≥2 ETs, and both LBs and failures/miscarriages
