# Supplementary material for: Quantitative Standardized Expansion Assay: An Artificial Intelligence-Powered Morphometric Description of Blastocyst Expansion and Zona Thinning Dynamics
Source: Life (Basel). 2024 Oct 30;14(11):1396. doi: 10.3390/life14111396 (PMC11595650; doi:10.3390/life14111396)
Supplement: Supplementary file 1 [file life-14-01396-s001.zip › Figure S6.pdf]

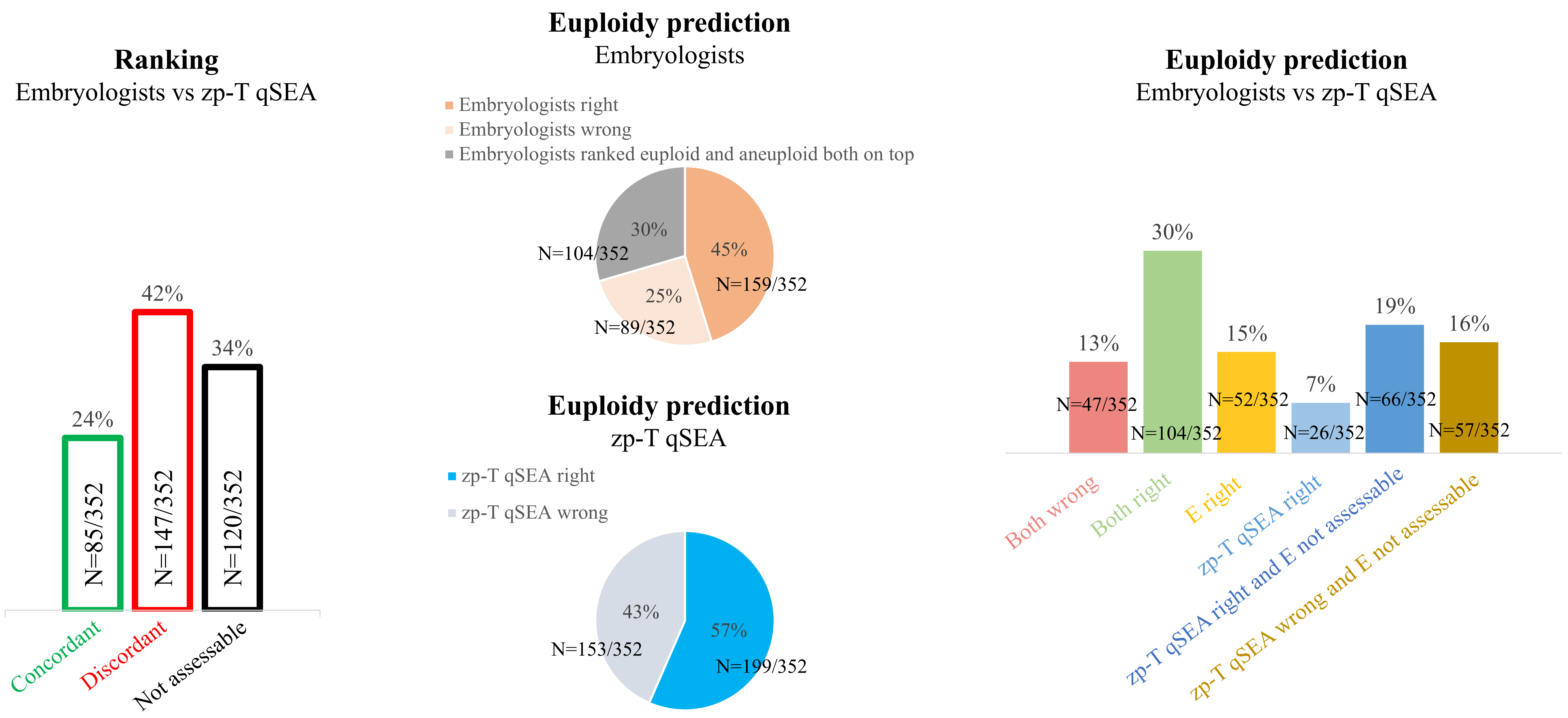
Ranking
Embryologists vs zp-T qSEA
24%
42%
34%
N=85/352
N=147/352
N=120/352
Concordant
Discordant
Not assessable
Euploidy prediction
Embryologists
Embryologists right
Embryologists wrong
Embryologists ranked euploid and aneuploid both on top
30%
N=104/352
45%
N=159/352
25%
N=89/352
Euploidy prediction
zp-T qSEA
zp-T qSEA right
zp-T qSEA wrong
43%
N=153/352
57%
N=199/352
Euploidy prediction
Embryologists vs zp-T qSEA
13%
N=47/352
30%
N=104/352
15%
N=52/352
7%
N=26/352
19%
N=66/352
16%
N=57/352
Both wrong
Both right
E right
zp-T qSEA right
zp-T qSEA right and E not assessable
zp-T qSEA wrong and E not assessable
