# Supplementary material for: Quantitative Standardized Expansion Assay: An Artificial Intelligence-Powered Morphometric Description of Blastocyst Expansion and Zona Thinning Dynamics
Source: Life (Basel). 2024 Oct 30;14(11):1396. doi: 10.3390/life14111396 (PMC11595650; doi:10.3390/life14111396)
Supplement: Supplementary file 1 [file life-14-01396-s001.zip › Figure S7.pdf]

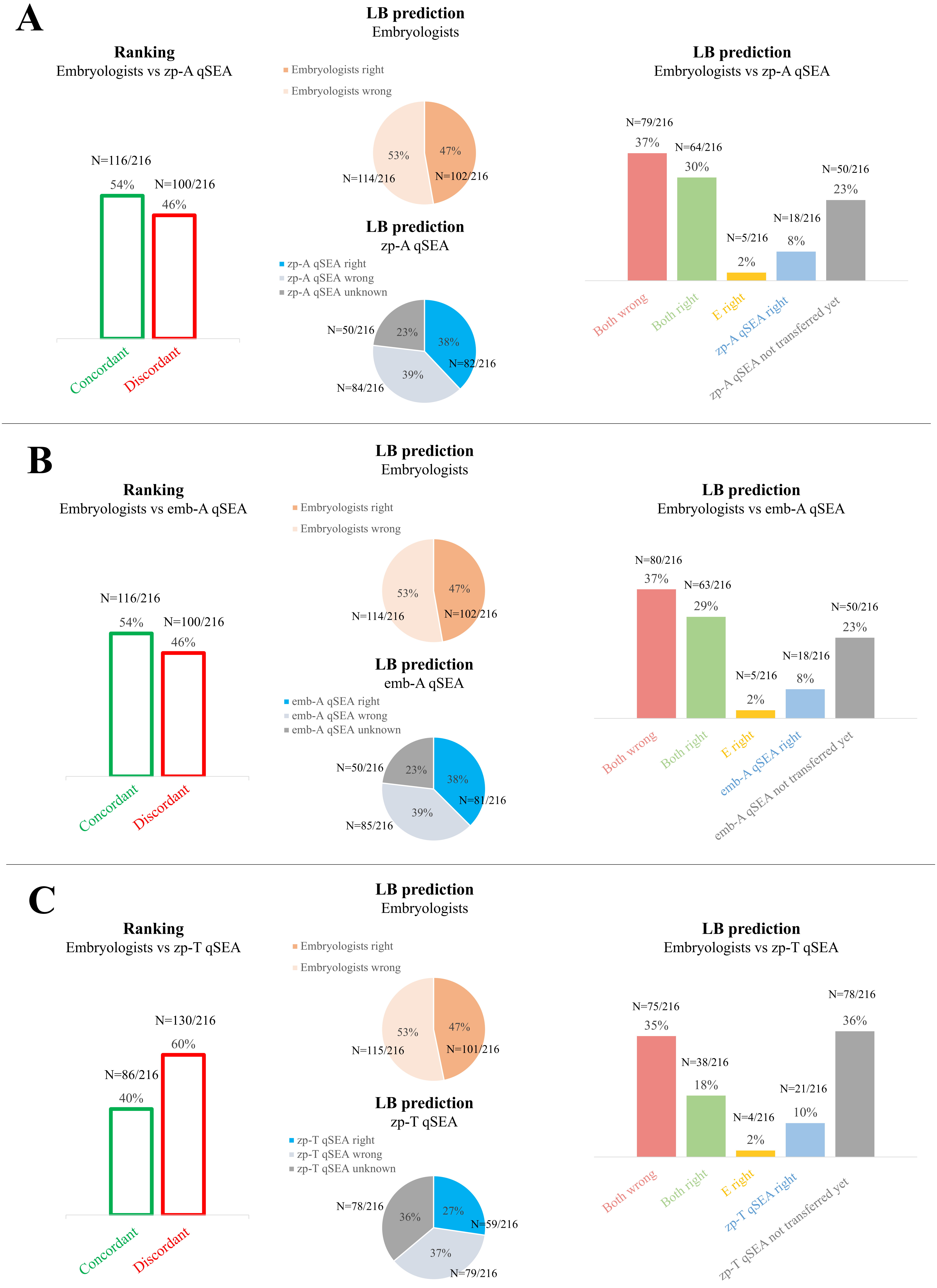
A
Ranking
Embryologists vs zp-A qSEA
N=116/216
54%
N=100/216
46%
Concordant
Discordant
LB prediction
Embryologists
Embryologists right
Embryologists wrong
53%
N=114/216
47%
N=102/216
LB prediction
zp-A qSEA
zp-A qSEA right
zp-A qSEA wrong
zp-A qSEA unknown
N=50/216
23%
38%
N=82/216
39%
N=84/216
LB prediction
Embryologists vs zp-A qSEA
N=79/216
37%
N=64/216
30%
N=5/216
2%
N=18/216
8%
N=50/216
23%
Both wrong
Both right
E right
zp-A qSEA right
zp-A qSEA not transferred yet
B
Ranking
Embryologists vs emb-A qSEA
N=116/216
54%
N=100/216
46%
Concordant
Discordant
LB prediction
Embryologists
Embryologists right
Embryologists wrong
53%
N=114/216
47%
N=102/216
LB prediction
emb-A qSEA
emb-A qSEA right
emb-A qSEA wrong
emb-A qSEA unknown
N=50/216
23%
38%
N=81/216
39%
N=85/216
LB prediction
Embryologists vs emb-A qSEA
N=80/216
37%
N=63/216
29%
N=5/216
2%
N=18/216
8%
N=50/216
23%
Both wrong
Both right
E right
emb-A qSEA right
emb-A qSEA not transferred yet
C
Ranking
Embryologists vs zp-T qSEA
N=130/216
60%
N=86/216
40%
Concordant
Discordant
LB prediction
Embryologists
Embryologists right
Embryologists wrong
53%
N=115/216
47%
N=101/216
LB prediction
zp-T qSEA
zp-T qSEA right
zp-T qSEA wrong
zp-T qSEA unknown
N=78/216
36%
27%
N=59/216
37%
N=79/216
LB prediction
Embryologists vs zp-T qSEA
N=75/216
35%
N=38/216
18%
N=4/216
2%
N=21/216
10%
N=78/216
36%
Both wrong
Both right
E right
zp-T qSEA right
zp-T qSEA not transferred yet
