# Supplementary material for: Quantitative Standardized Expansion Assay: An Artificial Intelligence-Powered Morphometric Description of Blastocyst Expansion and Zona Thinning Dynamics
Source: Life (Basel). 2024 Oct 30;14(11):1396. doi: 10.3390/life14111396 (PMC11595650; doi:10.3390/life14111396)
Supplement: Supplementary file 1 [file life-14-01396-s001.zip › Supplemental Tables 1_5.pdf]

Supplemental Table S1. Patients' characteristics

**Maternal age,** mean ± SD	38.9 ± 3.3 years old
**BMI,** mean ± SD	21.8 ± 3.6
**Previous conception(s),** N, %	
No	N=527/786, 67.0%
Yes	N=259/786, 33.0%
**Duration of infertility,** mean ± SD	3.4 ± 2.2 years
**Main cause of infertility,** N, %	
Idiopathic	N=524/786, 66.7%
Endocrine-ovulatory	N=20/786, 2.5%
Tubal	N=72/786, 9.2%
Endometriosis	N=56/786, 7.1%
Male factor	N=114/786, 14.5%
**Sperm quality**	
**No defects**	N=417/786, 53%
**1-2 defects**	N=238/786, 30%
**3 defects**	N=113/786, 14.3%
**Obstructive azoospermia**	N=10/786, 1.2%
**Non-obstructive azoospermia**	N=8/786, 1%

Supplemental Table S2. Distribution of the PGT-A cycles in the clinical simulation for the comparison of embryologists' (E) versus zp-T qSEA's performance in ranking euploid blastocysts on top. The results are summarized in Supplementary Figure S3.

** ≥2 embryos ranked on top according to the embryologists, that included also the embryo ranked on top according to the zp-T qSEA*

^ ≥2 embryos ranked on top according to the embryologists, of which ≥1 euploid and ≥1 aneuploid

**Ranking**	**E vs zp-T qSEA Concordant**		**E vs zp-T qSEA Discordant**						**E vs zp-T qSEA Not Assessable***			
**Euploidy prediction**	**Both wrong**	**Both right**	**Both wrong**	**Both right**	**E right**	**zp-T qSEA right**	**E not assessable^ but zp-T qSEA right**	**E not assessable^ but zp-T qSEA wrong**	**Both wrong**	**Both right**	**E not assessable^ but zp-T qSEA right**	**E not assessable^ but zp-T qSEA wrong**
	25	60	12	23	52	26	13	21	10	21	53	36

Supplemental Table S3. Distribution of the PGT-A cycles in the clinical simulation for the comparison of embryologists' (E) versus zp-A qSEA's performance in ranking euploid blastocysts that resulted in a live birth (LB) on top. The results are summarized in Supplementary Figure S4A.

Ranking (among euploid blastocysts)	E vs zp-A qSEA Concordant		E vs zp-A qSEA Discordant				
LB prediction (among euploid blastocysts)	Both wrong	Both right	Both wrong	Both right	E right	zp-A qSEA right	zp-A qSEA not transferred yet
	N=53 PGT-A cycles with ≥2 ETs, of which ≥1 LB and ≥1 failure						
	15	7	2	1	5	18	5
	N=99 PGT-A cycles with only 1 ET performed up to date						
	16	46	-	-	-	-	37
	N=14 PGT-A cycles with all transfers resulting in a LB						
	-	5	-	5	-	-	4
	N=50 PGT-A cycles with all transfers resulting in a failure						
	27	-	19	-	-	-	4

Supplemental Table S4. Distribution of the PGT-A cycles in the clinical simulation for the comparison of embryologists' (E) versus emb-A qSEA's performance in ranking euploid blastocysts that resulted in a live birth (LB) on top. The results are summarized in Supplementary Figure S4B.

Ranking (among euploid blastocysts)	E vs embA qSEA Concordant		E vs embA qSEA Discordant				
LB prediction (among euploid blastocysts)	Both wrong	Both right	Both wrong	Both right	E right	embA qSEA right	embA qSEA not transferred yet
	N=53 PGT-A cycles with ≥2 ETs, of which ≥1 LB and ≥1 failure						
	15	7	2	1	5	18	5
	N=99 PGT-A cycles with only 1 ET performed up to date						
	17	45	-	-	-	-	37
	N=14 PGT-A cycles with all transfers resulting in a LB						
	-	5	-	5	-	-	4
	N=50 PGT-A cycles with all transfers resulting in a failure						
	27	-	19	-	-	-	4

Supplemental Table S5. Distribution of the PGT-A cycles in the clinical simulation for the comparison of embryologists' (E) versus zp-T qSEA's performance in ranking euploid blastocysts that resulted in a live birth (LB) on top. The results are summarized in Supplementary Figure S4C.

**Ranking (among euploid blastocysts)**	**E vs zp-T qSEA Concordant**		**E vs zp-T qSEA Discordant**				
**LB prediction (among euploid blastocysts)**	**Both wrong**	**Both right**	**Both wrong**	**Both right**	**E right**	**zp-T qSEA right**	**zp-T qSEA not transferred yet**
	**N=53 PGT-A cycles with ≥2 ETs, of which ≥1 LB and ≥1 failure**						
	10	8	3	1	4	21	6
	**N=99 PGT-A cycles with only 1 ET performed up to date**						
	14	21	-	-	-	-	64
	**N=14 PGT-A cycles with all transfers resulting in a LB**						
	-	6	-	2	-	-	6
	**N=50 PGT-A cycles with all transfers resulting in a failure**						
	27	-	21	-	-	-	2
