# Supplementary figures and images for: Quantitative Standardized Expansion Assay: An Artificial Intelligence-Powered Morphometric Description of Blastocyst Expansion and Zona Thinning Dynamics
Source: Life (Basel). 2024 Oct 30;14(11):1396. doi: 10.3390/life14111396 (PMC11595650; doi:10.3390/life14111396)

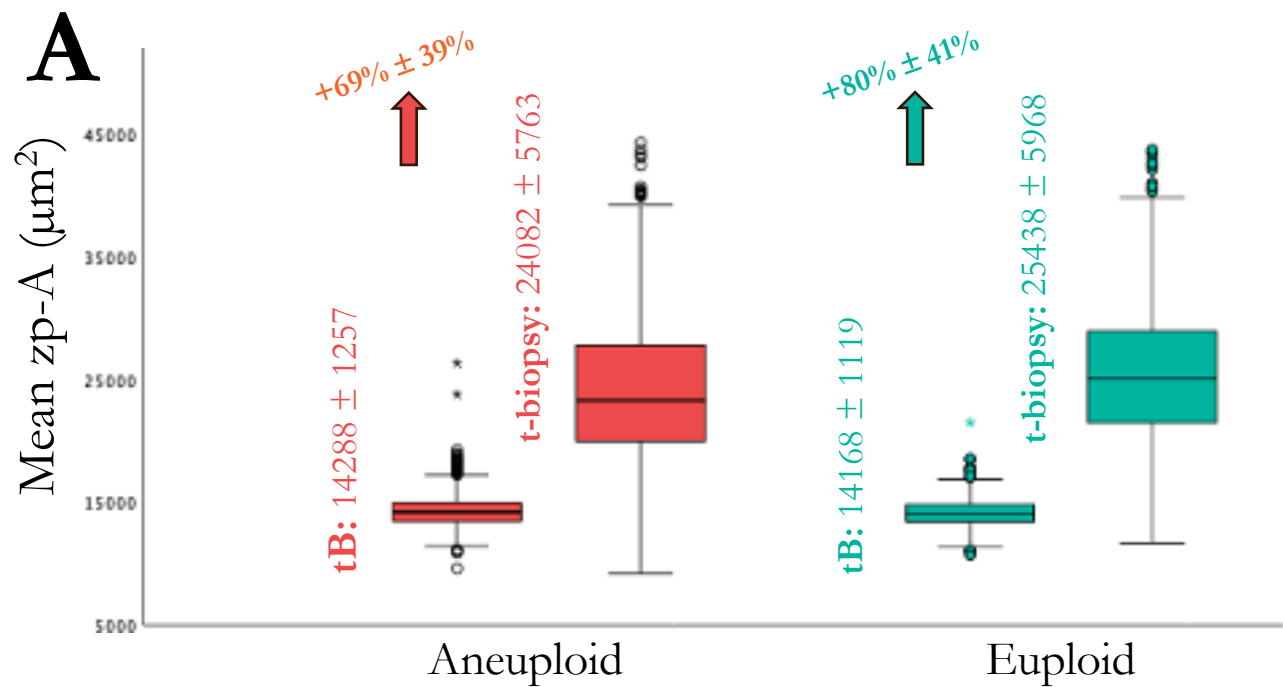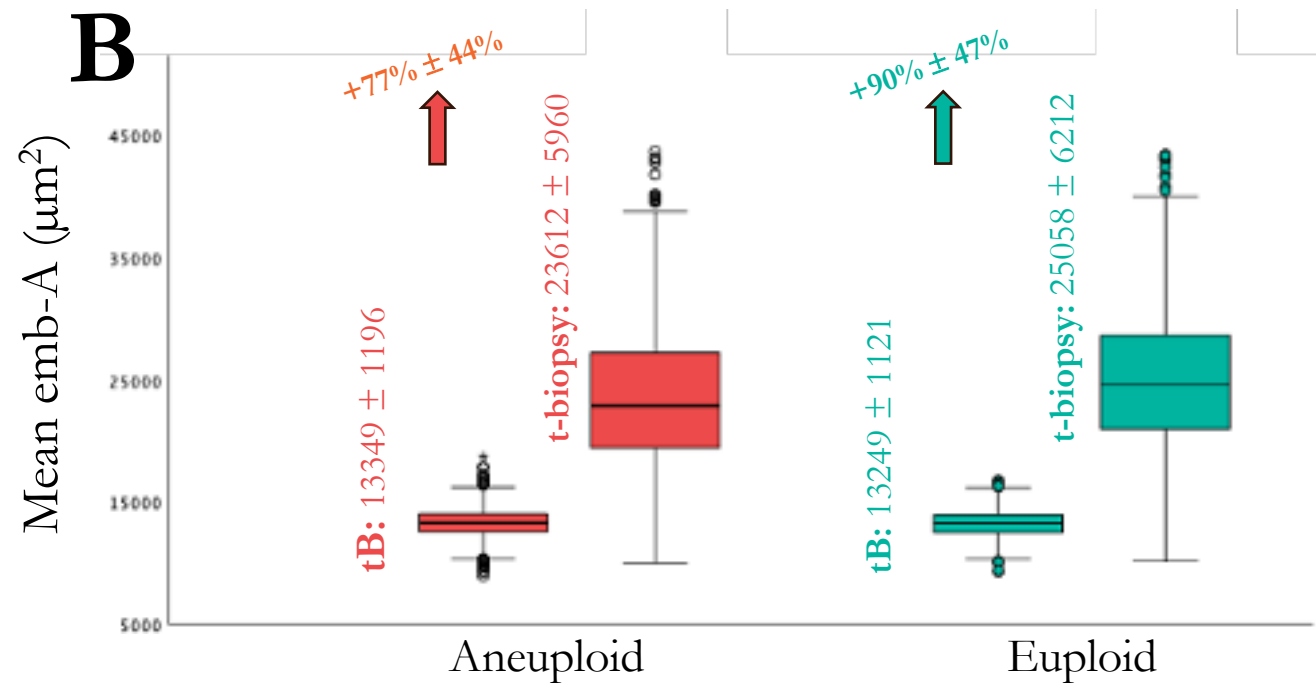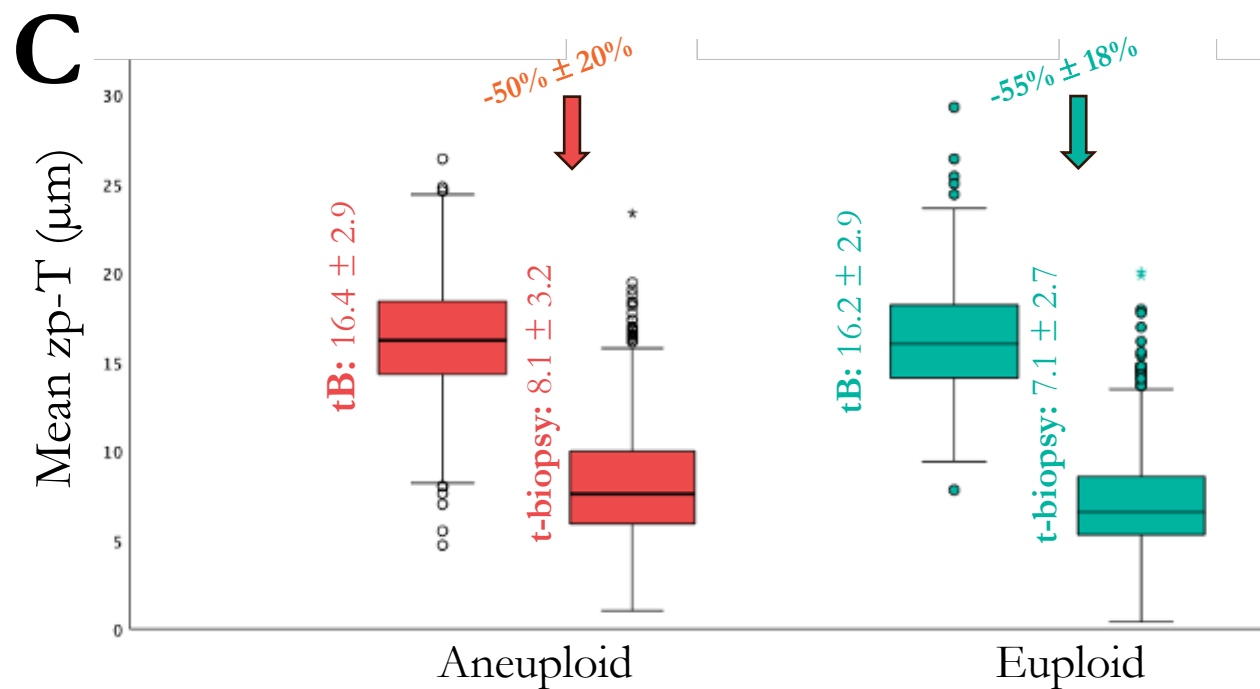

Supplement: Supplementary file 1 [file life-14-01396-s001.zip › Figure S2.pdf]

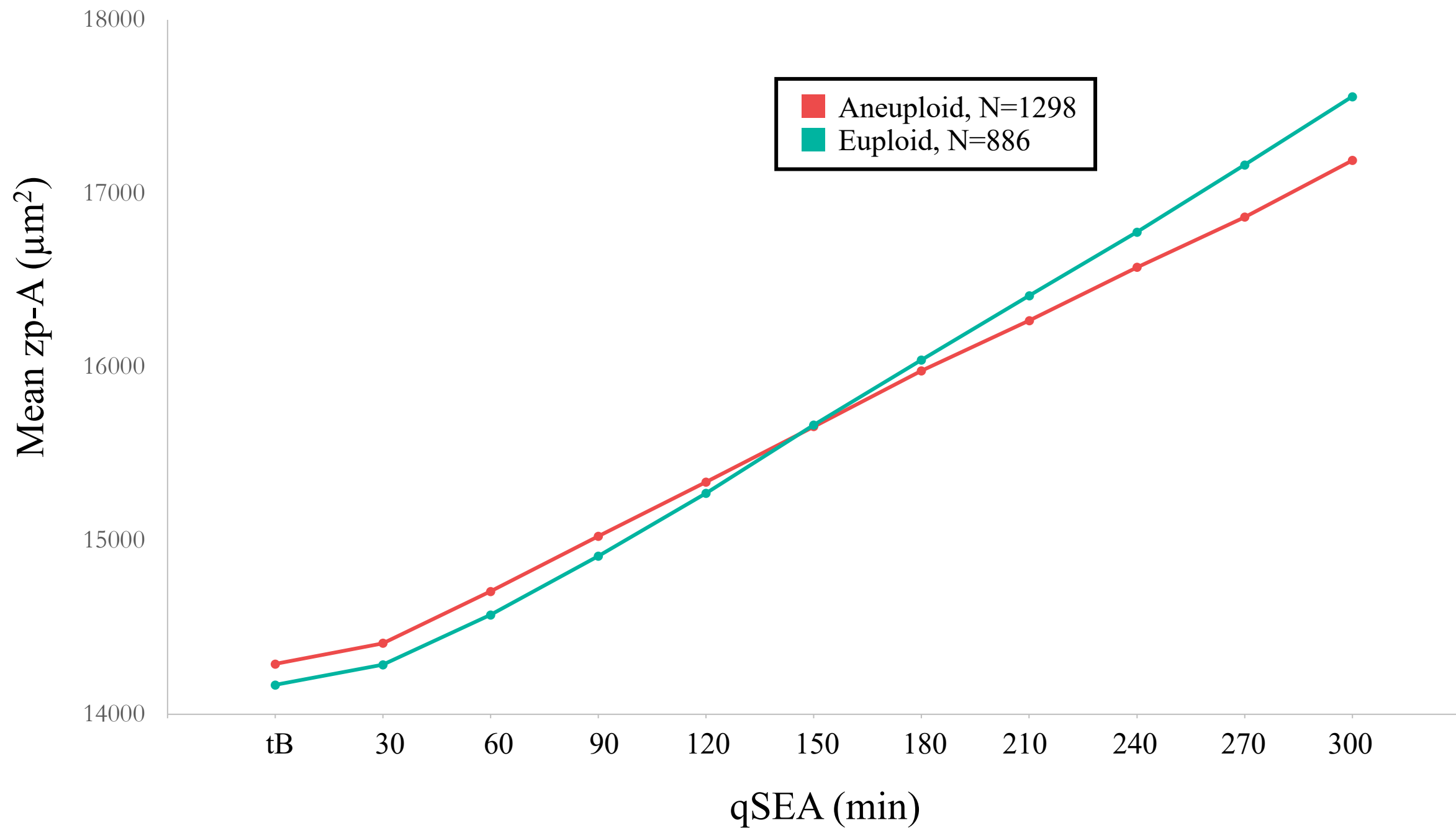

Supplement: Supplementary file 1 [file life-14-01396-s001.zip › Figure S3.pdf]

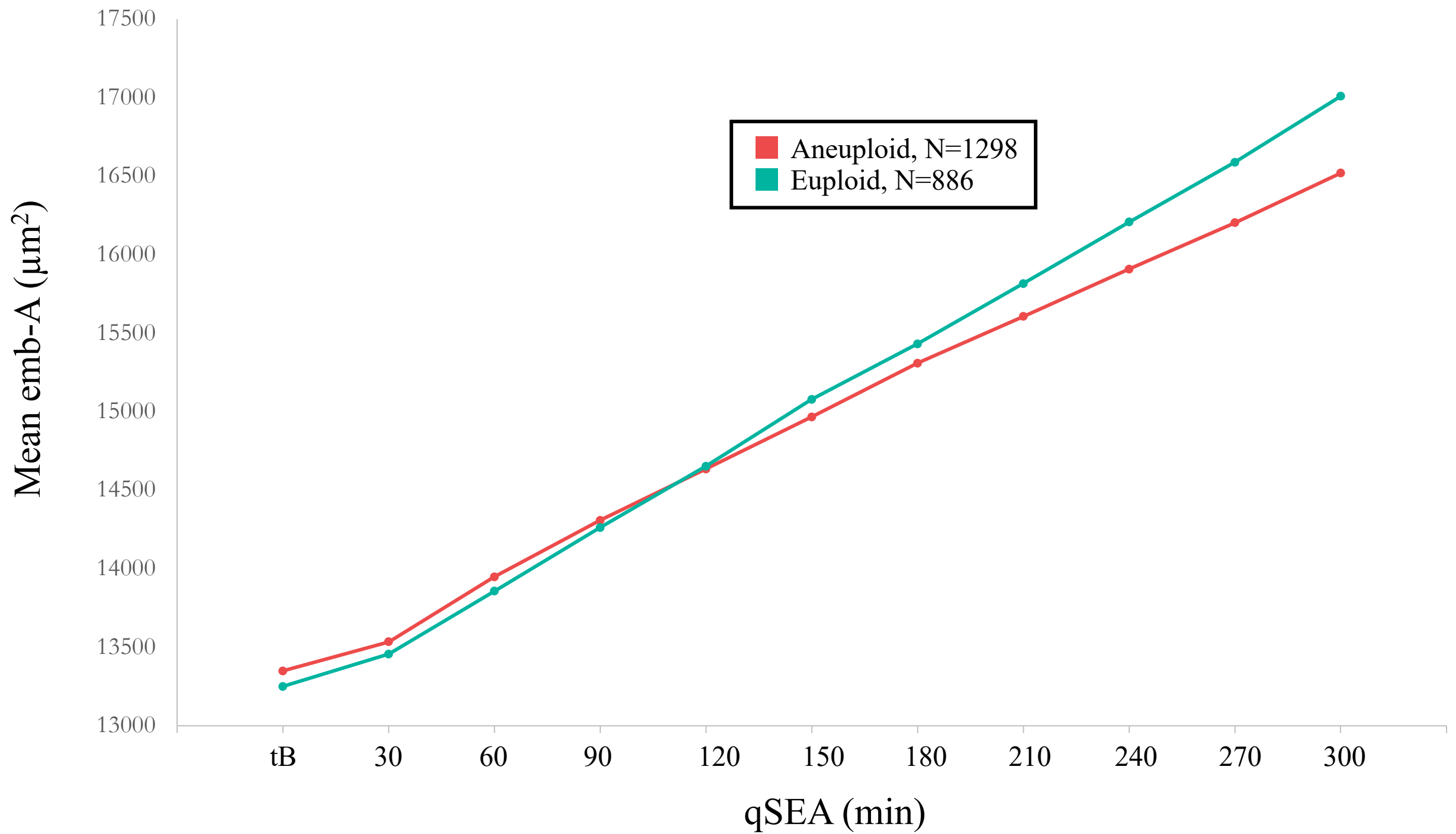

Supplement: Supplementary file 1 [file life-14-01396-s001.zip › Figure S4.pdf]

**A**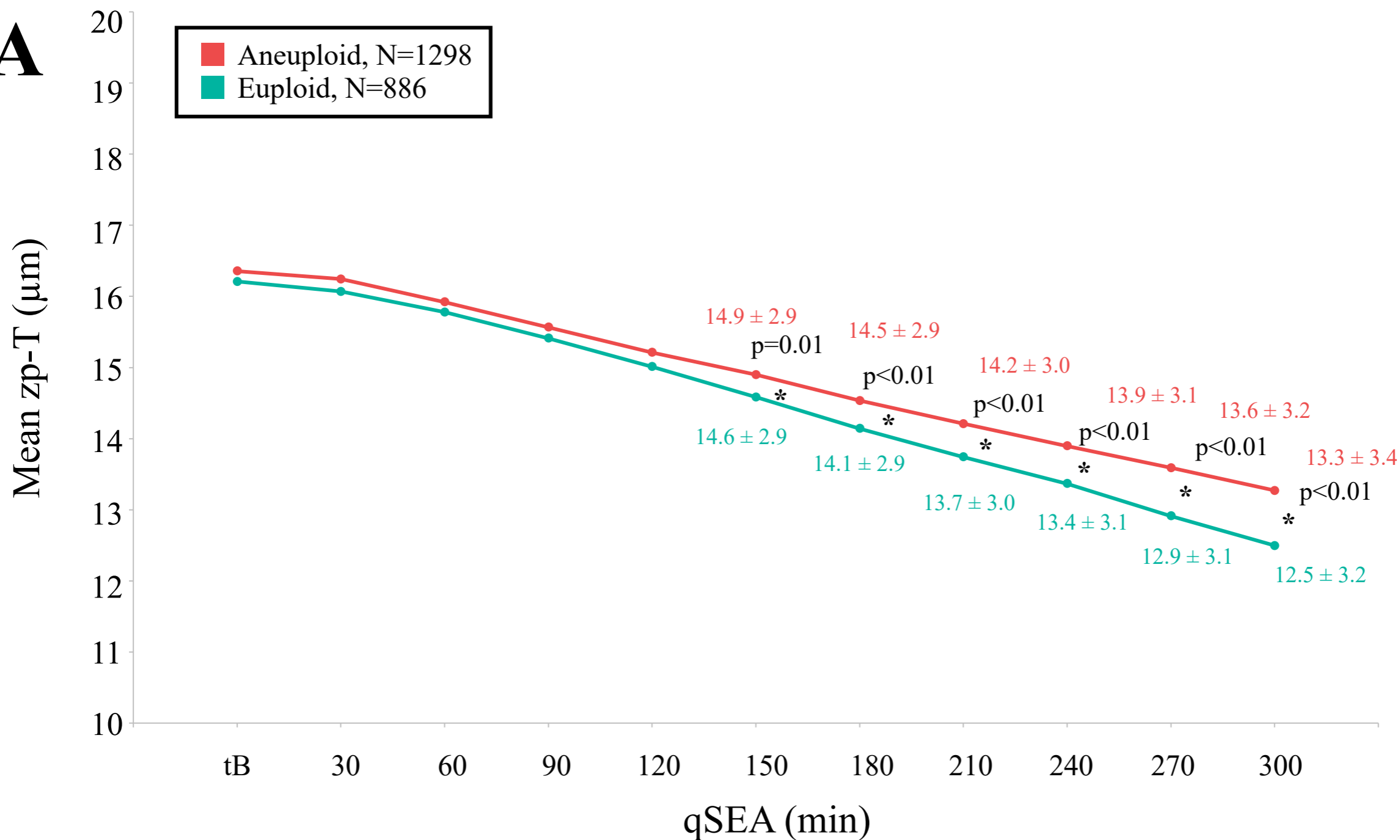**B**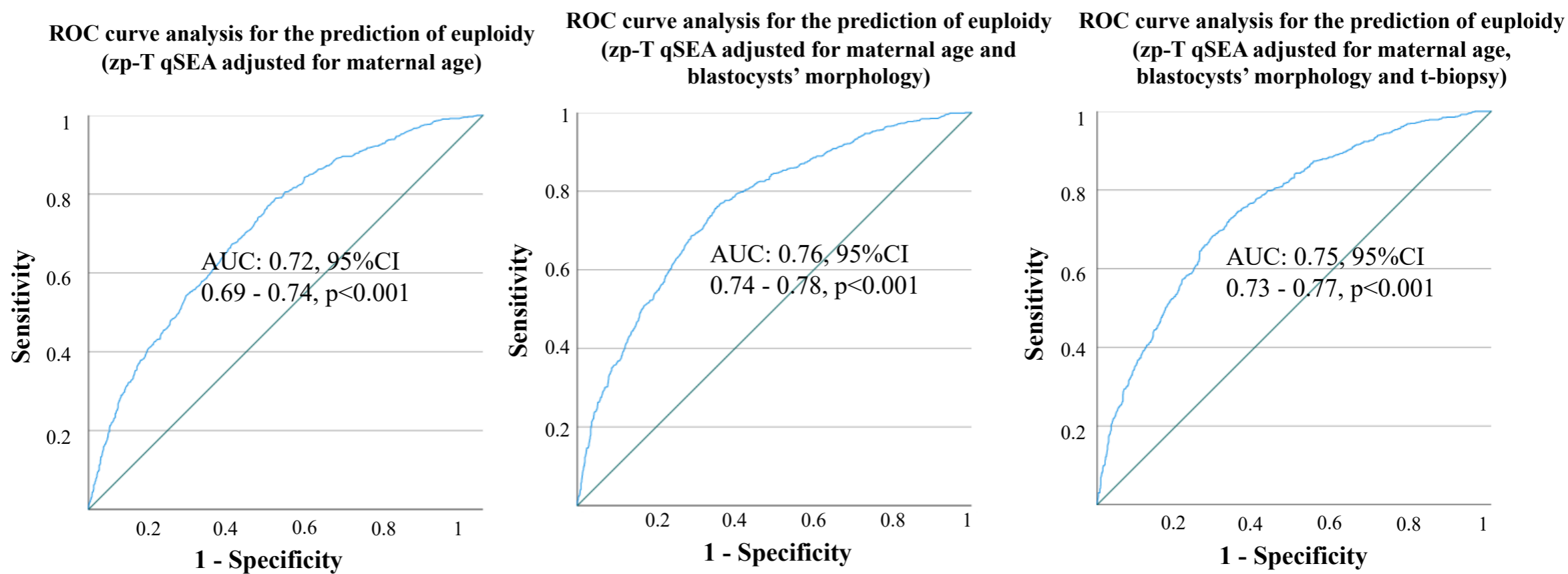

Supplement: Supplementary file 1 [file life-14-01396-s001.zip › Figure S5.pdf]
